# A Specific Reduction in Aβ_1−42_ vs. a Universal Loss of Aβ Peptides in CSF Differentiates Alzheimer's Disease From Meningitis and Multiple Sclerosis

**DOI:** 10.3389/fnagi.2018.00152

**Published:** 2018-05-24

**Authors:** Philipp Spitzer, Roland Lang, Timo J. Oberstein, Piotr Lewczuk, Natalia Ermann, Hagen B. Huttner, Ilias Masouris, Johannes Kornhuber, Uwe Ködel, Juan M. Maler

**Affiliations:** ^1^Department of Psychiatry and Psychotherapy, Friedrich-Alexander-University Erlangen-Nuremberg, University Hospital Erlangen, Erlangen, Germany; ^2^Institute of Clinical Microbiology, Immunology and Hygiene, Friedrich-Alexander-University Erlangen-Nuremberg, University Hospital Erlangen, Erlangen, Germany; ^3^Department of Neurodegeneration Diagnostics, Medical University of Bialystok, Bialystok, Poland; ^4^Department of Neurology, Friedrich-Alexander-University Erlangen-Nuremberg, University Hospital Erlangen, Erlangen, Germany; ^5^Department of Neurology, Ludwig-Maximilian-University, Munich, Germany

**Keywords:** meningitis, amyloid, Alzheimer's disease, multiple sclerosis, biomarker, dementia, neuroinflammation, neurodegeneration

## Abstract

A reduced concentration of Aβ_1−42_ in CSF is one of the established biomarkers of Alzheimer's disease. Reduced CSF concentrations of Aβ_1−42_ have also been shown in multiple sclerosis, viral encephalitis and bacterial meningitis. As neuroinflammation is one of the neuropathological hallmarks of Alzheimer's disease, an infectious origin of the disease has been proposed. According to this hypothesis, amyloid pathology is a consequence of a microbial infection and the resulting immune defense. Accordingly, changes in CSF levels of amyloid-β peptides should be similar in AD and inflammatory brain diseases. Aβ_1−42_ and Aβ_1−40_ levels were measured in cerebrospinal fluid by ELISA and Western blotting in 34 patients with bacterial meningitis (*n* = 9), multiple sclerosis (*n* = 5) or Alzheimer's disease (*n* = 9) and in suitable controls (*n* = 11). Reduced concentrations of Aβ_1−42_ were detected in patients with bacterial meningitis, multiple sclerosis and Alzheimer's disease. However, due to a concurrent reduction in Aβ_1−40_ in multiple sclerosis and meningitis patients, the ratio of Aβ_1−42_/Aβ_1−40_ was reduced only in the CSF of Alzheimer's disease patients. Urea-SDS-PAGE followed by Western blotting revealed that all Aβ peptide variants are reduced in bacterial meningitis, whereas in Alzheimer's disease, only Aβ_1−42_ is reduced. These results have two implications. First, they confirm the discriminatory diagnostic power of the Aβ_1−42_/Aβ_1−40_ ratio. Second, the differential pattern of Aβ peptide reductions suggests that the amyloid pathology in meningitis and multiple sclerosis differs from that in AD and does not support the notion of AD as an infection-triggered immunopathology.

## Introduction

In addition to amyloid plaque deposition, neuroinflammation is one of the neuropathological hallmarks of Alzheimer's disease (AD) (Heneka et al., 2015). Viral, bacterial and even fungal antigens have been found in association with the pathognomonic amyloid-beta (Aβ) depositions (Itzhaki, 2014; Little et al., 2014; Piacentini et al., 2015; Pisa et al., 2015; Zhan et al., 2016). The secretion of Aβ peptides during inflammation and the observation of anti-infective properties of Aβ peptides support the idea that the production of Aβ peptides provides an immune defense (Spitzer et al., 2010, 2016; Condic et al., 2014; Kumar et al., 2016). These and other findings have culminated in the formulation of the infection hypothesis of AD (Miklossy, 2011). According to this hypothesis, amyloid deposition is the consequence of ongoing neuroinflammation evoked by a pathogen that evades the immune system.

In the diagnosis of AD, decreased levels of soluble Aβ peptide 1-42 (Aβ_1−42_) in the cerebrospinal fluid (CSF) are widely accepted as a surrogate for brain amyloidosis (McKhann et al., 2011; Blennow and Zetterberg, 2015; Jack et al., 2016; Lewczuk et al., 2017). Fitting perfectly into the infection hypothesis, reduced levels of Aβ_42_ are also found in patients with brain infections, such as bacterial meningitis, herpes encephalitis or human immunodeficiency virus (HIV)-associated dementia (Sjögren et al., 2001; Krut et al., 2013). Aβ_1−42_ is generated during cleavage of amyloid precursor protein (APP). In addition to Aβ_42_, several other Aβ peptide variants with different C- and N-termini are generated during this process (Wiltfang et al., 2002). It is hypothesized that it is not the overproduction of Aβ_42_ that leads to its deposition but rather an imbalance of the different Aβ peptide variants (Hasegawa et al., 1999; Jan et al., 2008). Consequently, the concentration of Aβ_1−42_ in relation to Aβ_1−40_, the most abundant Aβ peptide variant, was found to be superior to Aβ_1−42_ alone as a biomarker for AD (Lewczuk et al., 2004, 2015a,b; Janelidze et al., 2016; Niemantsverdriet et al., 2017). If this imbalance between Aβ_42_ and Aβ_40_ that is observed in AD could also be found in inflammatory brain diseases, those data would further support the infection hypothesis of AD.

Therefore, this study investigates whether the changes in Aβ_1−42_ and Aβ_1−40_ levels in CSF during AD resemble those observed in multiple sclerosis (MS) and bacterial meningitis and whether the Aβ-ratio (Aβ_1−42_/Aβ_1−40_) differs between these diseases.

## Materials and methods

### Patients

Samples were collected in the memory clinic of the Department of Psychiatry, Erlangen, the Neurological Department, Erlangen, and the Neurological Department of Ludwig-Maximilian-University (LMU), Munich. Patients or their legal representatives provided their informed consent, and the study protocol was approved by the ethics committee of the University Hospital Erlangen-Nuremberg (project no. 3987) and of LMU Munich (project no. 349-15 and 174-11). Patients included in this study underwent psychiatric, neurological, medical and routine laboratory examinations. Additionally, an MRI scan and a CSF analysis were performed. The CSF of patients with meningitis was drawn under emergency conditions, so oligoclonal bands were not routinely determined. The diagnosis of AD was made according to the National Institute of Neurological and Communicative Disorders and Stroke and the Alzheimer's Disease and Related Disorders Association (NINCDS-ADRDA) criteria, taking into account the Aβ_42_/Aβ_40_ ratio and the total tau and phospho-tau levels in the CSF (Albert et al., 2011; McKhann et al., 2011). The Erlangen Score (ES) algorithm (Lewczuk et al., 2009, 2015a) was also used to classify patients: all AD patients had an ES ≥ 3, and all controls had an ES ≤ 1. In reference to the criteria suggested by Jack et al, the patients within the AD group were classified according to the presence (+) or absence (–) of Aβ (A), neurofibrillary tangles (T) and neurodegeneration (N) as A+/T+/N+, whereas those in the control group were A–/T–/N– (Jack et al., 2016). Amyloid pathology was evaluated by the Aβ_42_/Aβ_40_ ratio, tau pathology was evaluated by phospho-tau levels, and neurodegeneration was evaluated by total-tau levels and temporo-parietal atrophy in the MRI scan. Patients with intermediate signs of AD pathophysiology were excluded. MS was diagnosed according to the revised McDonald criteria (Polman et al., 2011), and meningitis was diagnosed according to the European Society of Clinical Microbiology and Infectious Diseases (ESCMID) guidelines (van de Beek et al., 2016). The control group comprised five patients with tension headache, one with schizophrenia, two with idiopathic epilepsy and one with a depressive episode. CSF samples were collected in polypropylene tubes, centrifuged within 24 h after sampling and stored in aliquots at −80°C until further use.

### ELISA

Aβ_1−40_ and Aβ_1−42_ levels were quantified with certified ELISA tests (IBL international GmbH, Hamburg, Germany) according to the manufacturer's instructions. Samples were thawed immediately before the analysis and diluted 1:20 in reagent diluent. Then, 100 μl of each sample, standard, positive control or blank were added in duplicate to a pre-coated microtiter plate. After incubation for 2 h, the plate was washed, and horseradish peroxidase (HRP)-conjugated detection antibody (clone 82E1) was added for another 1 h. After washing, the chromogenic substrate 3,3′,5,5′-tetramethylbenzidine (TMB) was added, and the plate was read. The analyses were performed under careful quality control, and a measurement was regarded as valid if the range-to-average coefficient of the duplicate measurements was below 20%.

### SDS-PAGE/western blotting

The immunoprecipitation, urea-sodium dodecyl sulfate-polyacrylamide gel electrophoresis (SDS-PAGE) and Western blotting procedures have been described in detail before (Wiltfang et al., 2002; Oberstein et al., 2015). In short, immunoprecipitation was carried out with mouse anti-amyloid antibody 6E10 (Biolegend, formerly Covance, Koblenz, Germany), reactive to amino acids 1-16 of the Aβ peptide, covalently bound to magnetic sheep anti-mouse Dynabeads®; M-280 (10 mg/ml; Dynal, Hamburg, Germany). Samples were loaded onto bicine-SDS gels containing 8 M urea and separated for 55 min at 25 mA/gel. After semi-dry blotting onto polyvinylidene fluoride (PVDF)-membranes with a discontinuous buffer system, immunolabeling was performed with the anti Aβ_1−x_ antibody 82E1, which only recognized Aβ peptides starting with amino acid 1 (1:1,000 in phosphate-buffered saline-Tris (PBS-T); IBL, Hamburg Germany). ECL® (enhanced chemiluminescence) prime (GE Healthcare, Freiburg, Germany) was used to develop the immunoblots, and chemiluminescence was detected using an Amersham® Imager 600 instrument (GE Healthcare, Freiburg, Germany). The antibodies (6E10 for the immunoprecipitation and 82E1 for the detection) were chosen to achieve the highest possible sensitivity in this system.

### Statistical analysis

Statistical analysis was carried out with Prism® 6.0 (GraphPad Software Inc., La Jolla, CA, USA). Assuming a Gaussian distribution, parametric ANOVAs for independent samples followed by Fisher's *post hoc* tests were used to compare measurements among the different groups. To evaluate the diagnostic accuracy of the Aβ_1−42_/Aβ_1−40_ ratio, a receiver operating characteristic (ROC) curve was generated. Pearson's correlation coefficient (r) was calculated to investigate the associations between different analytes. The results are presented as the mean with standard deviations and were considered to be significant at a *p*-value < 0.05.

## Results

### Study population

Samples were collected according to the same standard operating procedures at the Department of Psychiatry, Erlangen, and the Neurology Departments in Erlangen and Munich. Due to the ages at which the different pathologies typically occur, the samples could not be matched for age. However, there was no statistically significant age difference between the patients with meningitis and those with AD or between the controls and the patients with MS. The patients with meningitis had increased peripheral leucocyte counts, increased levels of C-reactive protein (CRP) and CSF protein, cells and erythrocytes and a higher CSF/serum albumin ratio. Patients with MS had slightly increased CSF leucocyte counts and positive oligoclonal bands in the CSF (Table 1).

**Table 1:** Patient characteristics—Patient characteristics are presented as the mean (standard deviation).

	**Con**	**MS**	**Meningitis**	**AD**
N (female)	9 (3)	5 (4)	9 (5)	11 (6)
Age [years]	44.1 (19.7)	36.4 (4.6)	64.8 (19.3)	69.9 (11.2)
Blood leukocytes [/μl]	8.6 (4.1)	6.9 (1.6)	16.9 (7.1)	6.4 (1.7)
Serum CRP [mg/l]	6.6 (13.4)	1.8 (1.3)	227.3 (100.6)	2.2 (2.4)
OCB (+/−)	0/9	5/0	1/2	0/11
Total protein (CSF) [mg/l]	583.3 (113.9)	464.0 (166.1)	4500 (3430)	370.4 (109.7)
Albumin ratio	6.0 (2.2)	7.3 (2.8)	85.0 (70.0)	6.4 (2.1)
Leukocytes (CSF) [/μl]	1.4 (1.3)	22.6 (14.9)	9543 (9827)	1.4 (0.9)
Erythrocytes (CSF) [/μl]	5.0 (10.3)	1.5 (1.3)	1295 (2015)	11.2 (22.7)

*N, number; CON, control; MS, multiple sclerosis; AD, Alzheimer's disease; CRP, C-reactive protein; OCB, oligoclonal IgG bands on isoelectric focusing; CSF, cerebrospinal fluid*.

### Reduced Aβ_1−42_ in AD, MS and meningitis

**Figure 1:**
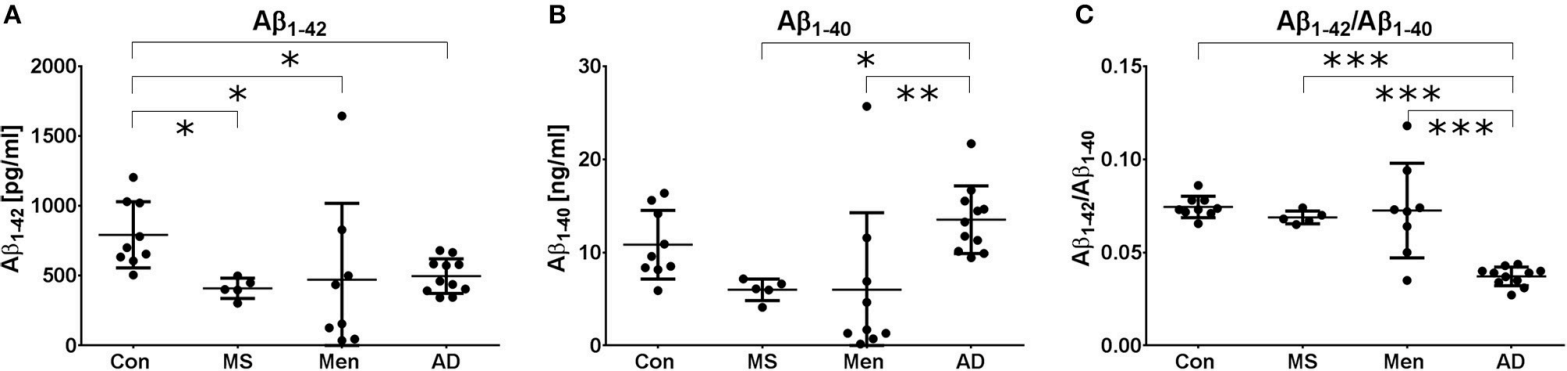
Different Aβ peptide profiles in AD, MS and bacterial meningitis Aβ_1−42_ **(A)** and Aβ_1−40_ **(B)** as well as the ratio (Aβ_1−42_/Aβ_1−40_) **(C)** were quantified by ELISA in the CSF of healthy controls (Con) and patients with multiple sclerosis (MS), meningitis (Men) and Alzheimer's disease (AD). The results are presented as the mean and standard deviation. Differences were calculated with ANOVA followed by the Fisher's *post hoc* test; ^*^p < 0.05, ^**^p < 0.01, ^***^p < 0.001.

All measurements were performed in the same laboratory to avoid inter-center variations. Aβ_1−40_ and Aβ_1−42_ levels were quantified by ELISA. One patient in the meningitis group had an Aβ_1−42_ concentration below the lower limit of quantification and was excluded from further analysis. As expected, a reduced concentration of Aβ_1−42_ was found in AD samples. Additionally, in MS and bacterial meningitis, concentrations of Aβ_1−42_ were reduced (Figure 1A). The concentration of Aβ_1−40_ also tended to be reduced in MS and meningitis but not in AD (Figure 1B). As a consequence, the ratio of Aβ_1−42_/Aβ_1−40_ was reduced only in AD (Figure 1C). No significant correlations between the increased values of erythrocytes, leukocytes or protein and the reduced concentrations of CSF Aβ_1−40_ or Aβ_1−42_ were found in patients with meningitis. The established threshold of 0.05 allowed for the differentiation of AD patient samples from control samples and samples from MS and meningitis patients with 100% sensitivity and 91% specificity.

### Reduced total amyloid in MS and meningitis but not AD

**Figure 2:**
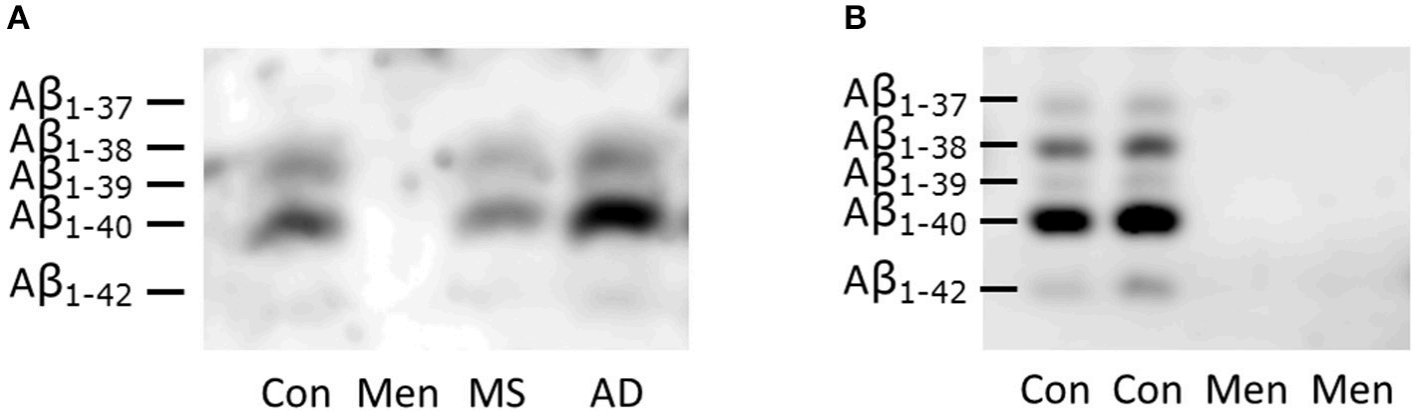
Reduced concentration of all Aβ peptides in bacterial meningitis **(A)** Representative SDS-PAGE/Western blots of directly loaded CSF from patients with bacterial meningitis (Men), multiple sclerosis (MS), Alzheimer's disease (AD) and controls (CON). **(B)** Immunoprecipitated samples from two representative patients with meningitis and two controls are depicted. The identities of the different Aβ peptide variants were defined in previous studies and are indicated on the left-hand side. Note that the longer peptides migrate below the shorter ones in this SDS-PAGE system containing urea.

CSF from representative patients with bacterial meningitis, multiple sclerosis and AD as well as one control was loaded directly onto SDS gels containing 8 M urea (Figure 2A). To control for artificially low concentrations of Aβ peptides due to the increased protein concentrations in meningitis, an additional separation of CSF from two patients with meningitis and two controls after immunoprecipitation with the 6E10 antibody was carried out (Figure 2B). The detection was performed with the anti-amyloid 82E1 antibody, which is specific for Aβ_1−x_ variants.

Reduced concentrations of all Aβ peptide variants were observed in the Western blots of CSF from meningitis patients (Figure 2). The concentration even dropped below the lower limit of detection.

## Discussion

The concentration of Aβ_1−42_ in CSF is reduced in patients with AD, MS and bacterial meningitis. However, the ratio of Aβ_1−42_/Aβ_1−40_ is reduced only in AD, while the concentrations of all Aβ peptide variants are similarly reduced in the CSF of patients with bacterial meningitis.

This is the first study to show the distinct changes in Aβ peptide variants in the CSF during AD compared to inflammatory brain diseases.

Nevertheless, the study has several limitations. The sample size is relatively small, and an exact matching of age and sex was not possible. Sample matching is nearly impossible for age because AD is usually diagnosed above the age of 60, while patients with MS become symptomatic before the age of 50. However, the CSF concentrations of Aβ peptides have been reported to be relatively stable throughout life and to be independent of sex in healthy subjects (Resnick et al., 2015).

To reduce the possible center effects, a balanced composition of the control and the meningitis groups was achieved. However, the samples for the MS group were all collected in Munich, while the samples for the AD group were all collected in Erlangen. However, the preanalytical handling of the samples was identical at all sites, and measurement of the Aβ peptides by ELISA was carried out in a single laboratory, as was recommended to effectively reduce center effects (Mattsson et al., 2013).

While Aβ_1−42_ is reduced in all three diseases, the observation that the Aβ ratio is reduced in AD but not in meningitis or MS points to a better diagnostic performance of the Aβ ratio. This improved performance has also been previously shown for the differential diagnosis of AD vs. non-neurodegenerative neuropsychiatric diseases, Lewy-body dementia, Parkinson's disease dementia or vascular dementia (Lewczuk et al., 2015b; Janelidze et al., 2016). Several others have therefore suggested to replace the measurement of Aβ_1−42_ with the Aβ ratio for AD diagnosis (Blennow and Zetterberg, 2015). Our study suggests that the Aβ ratio not only is able to increase the diagnostic performance in the differential diagnosis of neurodegenerative diseases but also helps to differentiate AD from neuroinflammatory diseases.

In the context of the infection hypothesis of AD, the observed differences among AD, MS and meningitis point to different pathophysiological processes behind the changes in CSF Aβ_1−42_ concentrations. While the processing of APP in AD is changed in a way that leads to an imbalance between the different Aβ peptide variants and allows the accumulation of Aβ_1−42_ into amyloid plaques, it was hypothesized that APP processing is impeded during CNS infections (Krut et al., 2013). Reduced concentrations of secreted (s)APPα and sAPPβ during meningitis support this interpretation (Krut et al., 2013). An alternative explanation is that serum proteins entering the CSF after breakdown of the blood brain barrier in meningitis might mask the detection of Aβ peptides. However, others have found no differences in Aβ peptide concentrations via ELISA after adding proteins to the CSF (Bjerke et al., 2010), and our data show no correlation between increased CSF protein concentrations and reduced concentrations of Aβ peptides. Additionally, we performed immunoprecipitation to release the Aβ peptides from their protein bonds. We suggest that the amyloid peptides build complexes around the invading pathogens and are therefore no longer measurable in the CSF (Spitzer et al., 2016). By agglutinating microorganisms and exerting a direct antimicrobial activity, Aβ peptides have been shown to accumulate around microorganisms *in vitro* (Soscia et al., 2010; Torrent et al., 2012; Kumar et al., 2016; Spitzer et al., 2016). The increased expression and distinct processing of APP during immunological activation *in vitro* also favors the idea of an immunological function and an increased consumption of Aβ peptides during infections and neuroinflammation in MS (Maler et al., 2008; Sondag and Combs, 2010; Spitzer et al., 2010). Further research is necessary to analyze the composition and fate of the Aβ peptides accumulating around the invading pathogens.

Current knowledge of inflammation-induced changes in Aβ peptide metabolism is mainly based on studies of acute infections (Spitzer et al., 2010; Kumar et al., 2016). Although MS is not an infectious disease, we used samples from MS patients to investigate the Aβ peptide levels in a chronic inflammatory disease. Mattsson and his colleagues measured the levels of Aβ_38_, Aβ_40_, and Aβ_42_ in patients with chronic neuroborreliosis (Mattsson et al., 2010). In both studies investigating two different entities causing chronic neuroinflammation, the concentrations of all measured Aβ peptide variants were reduced (Mattsson et al., 2010). Therefore, it seems that chronic neuroinfection and neuroinflammation are also associated with reduced levels of all Aβ peptides.

Taken together, the results of this study point to different mechanisms resulting in reduced Aβ_1−42_ levels in the CSF of patients with AD, bacterial meningitis or MS. The Aβ_1−42_/Aβ_1−40_ ratio may help to distinguish AD from neuroinflammatory diseases. Further studies are needed to confirm these findings.

## Author contributions

PS, RL, UK, PL, NE, and JM designed the study. PS, JK, TO, PL, UK, HH, and JM investigated the patients and collected the samples. PS, PL, TO, and NE carried out the experiments. The statistics were carried out by PS. PS and JM drafted the manuscript. All authors critically reviewed the manuscript and provided constructive comments to improve the quality of the manuscript. All authors have read and approved the final manuscript.

### Conflict of interest statement

PL received consultation and lectures honoraria from Innogenetics, IBL International, AJ Roboscreen, and Roche. The other authors declare that the research was conducted in the absence of any commercial or financial relationships that could be construed as a potential conflict of interest.

## References

[B1] Albert M. S. DeKosky S. T. Dickson D. Dubois B. Feldman H. H. Fox N. C.. (2011). The diagnosis of mild cognitive impairment due to Alzheimer's disease: recommendations from the National Institute on Aging-Alzheimer's Association workgroups on diagnostic guidelines for Alzheimer's disease. Alzheimers Dement. 7, 270–279. 10.1016/j.jalz.2011.03.008 21514249 PMC3312027

[B2] Bjerke M. Portelius E. Minthon L. Wallin A. Anckarsater H. Anckarsater R.. (2010). Confounding factors influencing amyloid Beta concentration in cerebrospinal fluid. Int. J. Alzheimers. Dis. 2010:986310. 10.4061/2010/986310 20798852 PMC2925386

[B3] Blennow K. Zetterberg H. (2015). The past and the future of Alzheimer's disease CSF biomarkers-a journey toward validated biochemical tests covering the whole spectrum of molecular events. Front. Neurosci. 9:345. 10.3389/fnins.2015.00345 26483625 PMC4586276

[B4] Condic M. Oberstein T. J. Herrmann M. Reimann M. C. Kornhuber J. Maler J. M.. (2014). N-truncation and pyroglutaminylation enhances the opsonizing capacity of Abeta-peptides and facilitates phagocytosis by macrophages and microglia. Brain Behav. Immun. 41, 116–25. 10.1016/j.bbi.2014.05.003 24876064

[B5] Hasegawa K. Yamaguchi I. Omata S. Gejyo F. Naiki H. (1999). Interaction between A β(1-42) and A β(1-40) in Alzheimer's beta-amyloid fibril formation *in vitro*. Biochemistry 38, 15514–15521 10569934 10.1021/bi991161m

[B6] Heneka M. T. Carson M. J. El Khoury J. Landreth G. E. Brosseron F. Feinstein D. L.. (2015). Neuroinflammation in Alzheimer's disease. Lancet Neurol. 14, 388–405. 10.1016/S1474-4422(15)70016-5 25792098 PMC5909703

[B7] Itzhaki R. F. (2014). Herpes simplex virus type 1 and Alzheimer's disease: increasing evidence for a major role of the virus. Front. Aging Neurosci. 6:202. 10.3389/fnagi.2014.00202 25157230 PMC4128394

[B8] Jack C. R. Jr. Bennett D. A. Blennow K. Carrillo M. C. Feldman H. H. Frisoni G. B.. (2016). A/T/N: An unbiased descriptive classification scheme for Alzheimer disease biomarkers. Neurology 87, 539–547. 10.1212/WNL.0000000000002923 27371494 PMC4970664

[B9] Jan A. Gokce O. Luthi-Carter R. Lashuel H. A. (2008). The ratio of monomeric to aggregated forms of Aβ40 and Aβ42 is an important determinant of amyloid-beta aggregation, fibrillogenesis, and toxicity. J. Biol. Chem. 283, 28176–28189. 10.1074/jbc.M803159200 18694930 PMC2661389

[B10] Janelidze S. Zetterberg H. Mattsson N. Palmqvist S. Vanderstichele H. Lindberg O.. (2016). CSF Aβbeta42/Aβbeta40 and Aβbeta42/Aβbeta38 ratios: better diagnostic markers of Alzheimer disease. Annals Clin. Transl. Neurol. 3, 154–165. 10.1002/acn3.274 27042676 PMC4774260

[B11] Krut J. J. Zetterberg H. Blennow K. Cinque P. Hagberg L. Price R. W.. (2013). Cerebrospinal fluid Alzheimer's biomarker profiles in CNS infections. J. Neurol. 260, 620–626. 10.1007/s00415-012-6688-y 23052602

[B12] Kumar D. K. Choi S. H. Washicosky K. J. Eimer W. A. Tucker S. Ghofrani J.. (2016). Amyloid-βbeta peptide protects against microbial infection in mouse and worm models of Alzheimer's disease. Sci. Transl. Med. 8, 340ra72. 10.1126/scitranslmed.aaf1059 27225182 PMC5505565

[B13] Lewczuk P. Esselmann H. Otto M. Maler J. M. Henkel A. W. Henkel M. K.. (2004). Neurochemical diagnosis of Alzheimer's dementia by CSF Aβbeta42, Aβbeta42/Aβbeta40 ratio and total tau. Neurobiol. Aging 25, 273–281. 10.1016/S0197-4580(03)00086-1 15123331

[B14] Lewczuk P. Kornhuber J. German Dementia Competence N. Toledo J. B. Trojanowski J. Q. Knapik-Czajka M.. (2015a). Validation of the erlangen score algorithm for the prediction of the development of Dementia due to Alzheimer's disease in Pre-Dementia Subjects. J. Alzheimers. Dis. 48, 433–441. 10.3233/JAD-150342 26402007 PMC5127395

[B15] Lewczuk P. Lelental N. Spitzer P. Maler J. M. Kornhuber J. (2015b). Amyloid-beta 42/40 cerebrospinal fluid concentration ratio in the diagnostics of Alzheimer's disease: validation of two novel assays. J. Alzheimers Dis. 43, 183–191. 10.3233/JAD-140771 25079805

[B16] Lewczuk P. Riederer P. O'Bryant S. E. Verbeek M. M. Dubois B. Visser P. J.. (2017). Cerebrospinal fluid and blood biomarkers for neurodegenerative dementias: an update of the consensus of the task force on biological markers in psychiatry of the world federation of societies of biological psychiatry. World J. Biol. Psychiatry 19, 244–328. 10.1080/15622975.2017.1375556 29076399 PMC5916324

[B17] Lewczuk P. Zimmermann R. Wiltfang J. Kornhuber J. (2009). Neurochemical dementia diagnostics: a simple algorithm for interpretation of the CSF biomarkers. J. Neural. Transm. 116, 1163–1167. 10.1007/s00702-009-0277-y 19653063

[B18] Little C. S. Joyce T. A. Hammond C. J. Matta H. Cahn D. Appelt D. M.. (2014). Detection of bacterial antigens and Alzheimer's disease-like pathology in the central nervous system of BALB/c mice following intranasal infection with a laboratory isolate of *Chlamydia pneumoniae*. Front. Aging Neurosci. 6:304. 10.3389/fnagi.2014.00304 25538615 PMC4257355

[B19] Maler J. M. Spitzer P. Klafki H. W. Esselmann H. Bibl M. Lewczuk P.. (2008). Adherence-dependent shifts in the patterns of beta-amyloid peptides secreted by human mononuclear phagocytes. Brain Behav. Immun. 22, 1044–1048. 10.1016/j.bbi.2008.04.003 18511234

[B20] Mattsson N. Andreasson U. Persson S. Carrillo M. C. Collins S. Chalbot S.. (2013). CSF biomarker variability in the Alzheimer's association quality control program. Alzheimers Dement. 9, 251–261. 10.1016/j.jalz.2013.01.010 23622690 PMC3707386

[B21] Mattsson N. Bremell D. Anckarsater R. Blennow K. Anckarsater H. Zetterberg H.. (2010). Neuroinflammation in Lyme neuroborreliosis affects amyloid metabolism. BMC Neurol. 10:51. 10.1186/1471-2377-10-51 20569437 PMC2902447

[B22] McKhann G. M. Knopman D. S. Chertkow H. Hyman B. T. Jack C. R. Jr. Kawas C. H.. (2011). The diagnosis of dementia due to Alzheimer's disease: recommendations from the National Institute on Aging-Alzheimer's Association workgroups on diagnostic guidelines for Alzheimer's disease. Alzheimers Dement. 7, 263–269. 10.1016/j.jalz.2011.03.005 21514250 PMC3312024

[B23] Miklossy J. (2011). Emerging roles of pathogens in Alzheimer disease. Expert Rev. Mol. Med. 13:e30. Epub 2011/09/22. 10.1017/S1462399411002006 21933454

[B24] Niemantsverdriet E. Ottoy J. Somers C. De Roeck E. Struyfs H. Soetewey F.. (2017). The cerebrospinal fluid Aβ1-42/Aβ1-40 ratio improves concordance with Amyloid-PET for diagnosing Alzheimer's disease in a clinical setting. J. Alzheimers. Dis. 60, 561–576. 10.3233/JAD-170327 28869470 PMC5611891

[B25] Oberstein T. J. Spitzer P. Klafki H. W. Linning P. Neff F. Knolker H. J.. (2015). Astrocytes and microglia but not neurons preferentially generate N-terminally truncated Abeta peptides. Neurobiol. Dis. 73, 24–35. 10.1016/j.nbd.2014.08.031 25204716

[B26] Piacentini R. Li Puma D. D. Ripoli C. Marcocci M. E. De Chiara G. Garaci E.. (2015). Herpes Simplex Virus type-1 infection induces synaptic dysfunction in cultured cortical neurons via GSK-3 activation and intraneuronal amyloid-beta protein accumulation. Sci. Rep. 5:15444. 10.1038/srep15444 26487282 PMC4614347

[B27] Pisa D. Alonso R. Rabano A. Rodal I. Carrasco L. (2015). Different brain regions are infected with fungi in Alzheimer's Disease. Sci. Rep. 5:15015. 10.1038/srep15015 26468932 PMC4606562

[B28] Polman C. H. Reingold S. C. Banwell B. Clanet M. Cohen J. A. Filippi M.. (2011). Diagnostic criteria for multiple sclerosis: 2010 revisions to the McDonald criteria. Ann. Neurol. 69, 292–302. 10.1002/ana.22366 21387374 PMC3084507

[B29] Resnick S. M. Bilgel M. Moghekar A. An Y. Cai Q. Wang M. C.. (2015). Changes in Abeta biomarkers and associations with APOE genotype in 2 longitudinal cohorts. Neurobiol Aging 36, 2333–2339. 10.1016/j.neurobiolaging.2015.04.001 26004017 PMC5084914

[B30] Sjögren M. Gisslen M. Vanmechelen E. Blennow K. (2001). Low cerebrospinal fluid beta-amyloid 42 in patients with acute bacterial meningitis and normalization after treatment. Neurosci. Lett. 314, 33–36. 10.1016/S0304-3940(01)02285-6 11698140

[B31] Sondag C. M. Combs C. K. (2010). Adhesion of monocytes to type I collagen stimulates an APP-dependent proinflammatory signaling response and release of Aβ1-40. J. Neuroinflammation 7:22. 10.1186/1742-2094-7-22 20302643 PMC2850892

[B32] Soscia S. J. Kirby J. E. Washicosky K. J. Tucker S. M. Ingelsson M. Hyman B.. (2010). The Alzheimer's disease-associated amyloid beta-protein is an antimicrobial peptide. PLoS ONE 5:e9505. 10.1371/journal.pone.0009505 20209079 PMC2831066

[B33] Spitzer P. Condic M. Herrmann M. Oberstein T. J. Scharin-Mehlmann M. Gilbert D. F.. (2016). Amyloidogenic amyloid-beta-peptide variants induce microbial agglutination and exert antimicrobial activity. Sci. Rep. 6:32228. 10.1038/srep32228 27624303 PMC5021948

[B34] Spitzer P. Herrmann M. Klafki H. W. Smirnov A. Lewczuk P. Kornhuber J.. (2010). Phagocytosis and LPS alter the maturation state of β-amyloid precursor protein and induce different Aβ peptide release signatures in human mononuclear phagocytes. J. Neuroinflammation 7:59. 10.1186/1742-2094-7-59 20929546 PMC2958903

[B35] Torrent M. Pulido D. Nogues M. V. Boix E. (2012). Exploring new biological functions of amyloids: bacteria cell agglutination mediated by host protein aggregation. PLoS Pathog. 8:e1003005. 10.1371/journal.ppat.1003005 23133388 PMC3486885

[B36] van de Beek D. Cabellos C. Dzupova O. Esposito S. Klein M. Kloek A. T.. (2016). ESCMID guideline: diagnosis and treatment of acute bacterial meningitis. Clin. Microbiol. Infect. 22(Suppl. 3), S37–S62. 10.1016/j.cmi.2016.01.007 27062097

[B37] Wiltfang J. Esselmann H. Bibl M. Smirnov A. Otto M. Paul S.. (2002). Highly conserved and disease-specific patterns of carboxyterminally truncated Aβ peptides 1-37/38/39 in addition to 1-40/42 in Alzheimer's disease and in patients with chronic neuroinflammation. J. Neurochem. 81, 481–496. 10.1046/j.1471-4159.2002.00818.x 12065657

[B38] Zhan X. Stamova B. Jin L. W. DeCarli C. Phinney B. Sharp F. R. (2016). Gram-negative bacterial molecules associate with Alzheimer disease pathology. Neurology 87, 2324–2332. 10.1212/WNL.0000000000003391 27784770 PMC5135029

